# Behavior of Au Nanoparticles under Pressure Observed by In Situ Small-Angle X-ray Scattering

**DOI:** 10.1021/acsnano.2c10643

**Published:** 2022-12-16

**Authors:** Camino Martín-Sánchez, Ana Sánchez-Iglesias, José Antonio Barreda-Argüeso, Alain Polian, Luis M. Liz-Marzán, Fernando Rodríguez

**Affiliations:** † MALTA Consolider, Departamento CITIMAC, Facultad de Ciencias, University de Cantabria, Santander39005, Spain; ‡ Faculté des Sciences, Département de Chimie Physique, Université de Genève, 30 Quai Ernest-Ansermet, CH-1211Genève, Switzerland; § CIC biomaGUNE, Basque Research and Technology Alliance (BRTA), Paseo de Miramón 194, Donostia-San Sebastián20014, Spain; ∥ Synchrotron SOLEIL, L’Orme des Merisiers St.Aubin, BP48, 91192Gif-sur-Yvette, France; ⊥ Sorbonne Université, UMR CNRS 7590, Institut de Minéralogie de Physique des Matériaux et de Cosmochimie, IMPMC, 75005Paris, France; # Ikerbasque, Basque Foundation for Science, Bilbao43018, Spain; ∇ Centro de Investigación Biomédica en Red, Bioingeniería, Biomateriales y Nanomedicina (CIBER-BBN), Paseo de Miramón 194, Donostia-San Sebastián20014, Spain

**Keywords:** gold nanoparticles, high-pressure, small-angle X-ray scattering, aggregation, pressure-induced diffusion

## Abstract

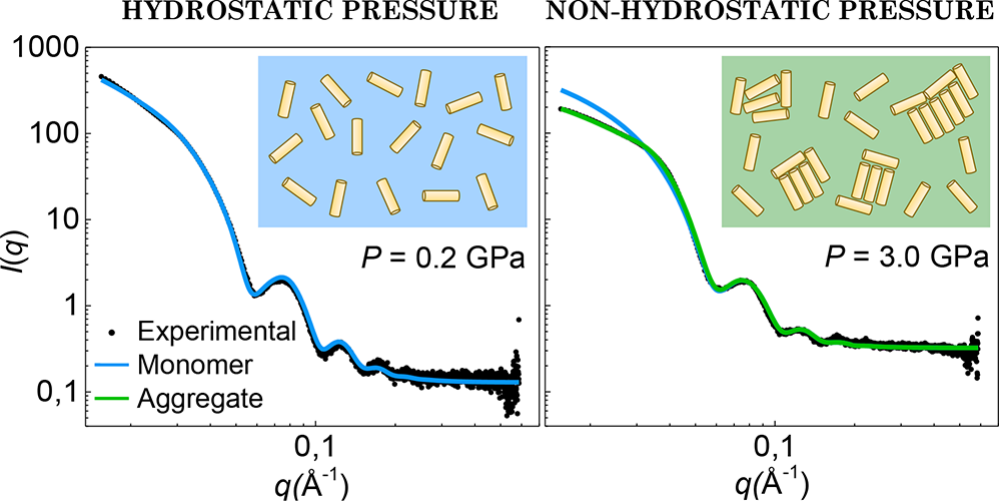

The mechanical properties and stability of metal nanoparticle colloids under high-pressure conditions are investigated by means of optical extinction spectroscopy and small-angle X-ray scattering (SAXS), for colloidal dispersions of gold nanorods and gold nanospheres. SAXS allows us to follow in situ the structural evolution of the nanoparticles induced by pressure, regarding both nanoparticle size and shape (form factor) and their aggregation through the interparticle correlation function *S*(*q*) (structure factor). The observed behavior changes under hydrostatic and nonhydrostatic conditions are discussed in terms of liquid solidification processes yielding nanoparticle aggregation. We show that pressure-induced diffusion and aggregation of gold nanorods take place after solidification of the solvent. The effect of nanoparticle shape on the aggregation process is additionally discussed.

## 1 Introduction

Metal nanoparticles have become one of the systems of choice as probes in high-pressure experiments. In particular, gold nanorods (AuNR) and nanospheres (AuNS) have turned out to be excellent model systems toward exploring pressure behavior of liquids, which also act as pressure transmitting media (PTM). Pressure dependences of the refractive index of water^1,2^ and some alcohols^3^ have been measured through localized surface plasmon resonance (LSPR) variations, using either AuNS or AuNR. Both the analytical expressions relating the LSPR and the refractive index of the surrounding medium, as well as the gold electron density and its optical parameters, are adequately described within the Gans theory.^4^ Vice versa, information on the gold nanoparticles (AuNP) themselves (deformation or electron density) can be inferred from plasmonic effects at high pressure, if the pressure dependence of the solvent—PTM—refractive index is known. Examples of this behavior have been reported elsewhere.^5,6^ In this way, high-pressure plasmonic sensing allows exploring the behavior of condensed systems (i.e. liquids like water, methane, and others), e.g., to mimic the environmental conditions attained in planet (and satellite) interiors.^7−9^ However, such important applications assume that the NP colloid remains stable within the whole pressure range, for an individual nanoparticle model to be applied. In this context, the use of high aspect ratio AuNR provides optimal sensitivity for refractive index sensing through their longitudinal LSPR (LLSPR); the higher the aspect ratio, the better the plasmonic sensitivity. However, AuNR, in contrast to AuNS, have a higher tendency to aggregate, and, if this is the case, then models based on pressure-induced LSPR shifts in nonaggregated single nanoparticles are no longer valid.

The plasmonic response of AuNP is exquisitely sensitive to changes in the shape and size of the NP, surrounding refractive index, and thus deformation and/or aggregation of the NPs. Even worse, the changes in the LSPR due to different effects can be quite similar to each other. Pressure-induced LSPR shifts of AuNS have been mainly associated with changes of the surrounding refractive index^1−3^ and the Au electron concentration in highly compressed NP^10−12^ as well as to reshaping into oblate deformations, particularly under nonhydrostatic pressure conditions.^13,14^ However, these effects are more intricate in AuNR due to their axial symmetry, making them more susceptible to deformation under nonhydrostatic compression, and to their greater tendency to form aggregates. The lack of reversibility of the AuNR extinction spectra in upstroke and downstroke supports this view.^6,15^ Consequently, an adequate interpretation of such structural changes through plasmonics requires confirmation by other complementary techniques, for the model to be validated. Transmission electron microscopy (TEM) is the most powerful tool to get direct information on the structural changes undergone by the NP. However, when using a diamond anvil cell (DAC) for performing a high-pressure experiment, TEM images can be obtained only before and after the pressure treatment (in recovered AuNP).^15^ In addition, whereas TEM is useful for studying variations of NP shape and size, it cannot visualize the NP distribution in the colloid because sample preparation will in general modify their state. Thus, in situ characterization of metal NP colloids at given pressure and temperature conditions remains experimentally unfeasible when utilizing DAC. In other words, obtaining in situ information on how the NP structure changes with pressure and whether the NP colloid remains stable under the application of pressure or undergoes aggregation and/or alloying is a difficult task that has only been recently achieved by means of optical spectroscopy under the mentioned intrinsic limitations.

**Figure 1:**
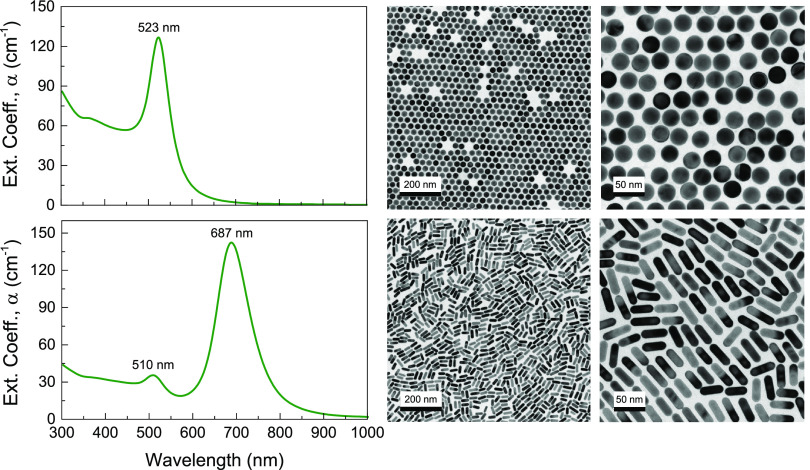
Optical extinction spectra and representative TEM images at different magnifications of the nanoparticles used in the experiments: 28.2 nm AuNS with [Au] = 11.0 mM, and [NS] = 9.7 × 10^12^ cm^–3^ (top row); and 39.1 × 13.1 nm^2^; *AR* = 3.0 AuNR with [Au] = 5.8 mM, and [NR] = 1.1 × 10^13^ cm^–3^ (bottom row).

Here we present a correlation study between plasmonics and colloid structure (NP shape, size, and aggregation/deformation) monitored through small-angle X-ray scattering (SAXS). Although limited in reachable pressure due to current technical constraints, SAXS provides a powerful tool for the in situ observation of the form factor (nanoparticle) and the structure factor (interparticle correlation). Hence, correlated SAXS and optical spectroscopy should allow us to describe a compact structural scenario on the effects of pressure in relatively dilute AuNP colloids ([AuNR] = 1.1 × 10^13^ cm^–3^; [AuNS] = 9.7 × 10^12^ cm^–3^) using ethanol (EtOH) as the solvent. Figure 1 shows the optical extinction spectra and TEM images of the spheres and rods utilized in the pressure experiments in this work. SAXS measurements provide information on the variation of size and shape of the AuNP as well as on their aggregation as a function of pressure, both in upstroke and downstroke. Interestingly, high-pressure SAXS has been previously applied to the study of assembled NP superstructures.^16−18^

## 2 Results

### 2.1 Optical Extinction Spectroscopy of Gold Colloids at High Pressure

**Figure 2:**
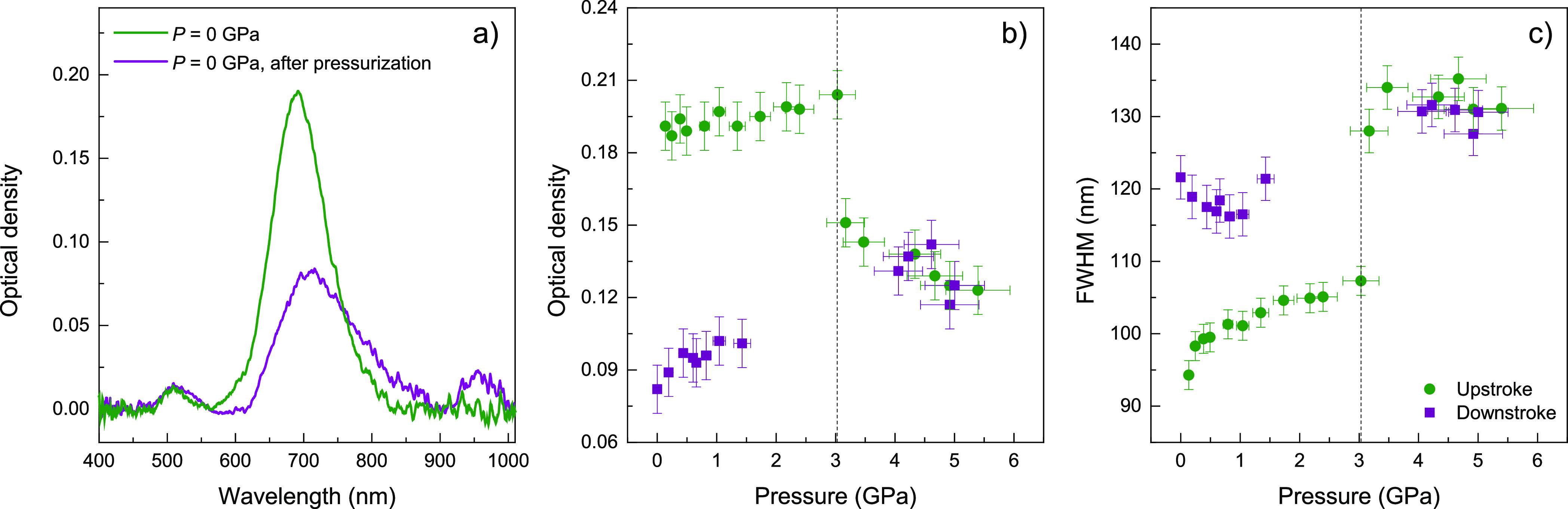
(a) Extinction spectra of 13 nm-diameter and *AR* = 3 AuNR in EtOH, measured at ambient pressure before and after pressurization at 5 GPa. LLSPR broadening and a new band at 960 nm are observed in the spectrum of the recovered AuNR colloid after pressure treatment. (b) Optical density at the LLSPR as a function of applied pressure. (c) Pressure dependence of the LLSPR fwhm obtained from optical extinction spectra of the AuNR dispersion. Filled circles (green) and squares (purple) correspond to upstroke and downstroke experimental data, respectively. Error bars indicate twice the standard deviation of each spectral parameter as derived by fitting the LLSPR band to a Log-Normal function. Vertical dashed lines indicate the hydrostaticity limit of the pressure-transmitting medium, determined from ruby probes.^23,24^

The variations in the extinction spectra of 13 nm-diameter and aspect ratio *AR* = 3 AuNR in EtOH with pressure in the 0–5 GPa range, both upstroke and downstroke, are shown in Figure 2. The comparison of the spectra measured at ambient pressure, before and after pressure treatment at 5 GPa, clearly indicates irreversible changes in the plasmonic response of the colloid. The red-shift, band broadening, and appearance of an additional LSPR band around 960 nm suggest AuNR aggregation after the application of pressure. It should be noted that this spectral difference is not observed in AuNS colloids, which display reversible plasmonic spectral features, even after pressure treatments up to 30 GPa.^15^ The deviation of the measured optical extinction at the LLSPR peak, *I*(*P*), with respect to that in the hydrostatic regime is also significant (see Figure 2). As expected, we observe an increase of *I*(*P*) with pressure, mainly due to a corresponding increase in the solvent refractive index under hydrostatic conditions. However, *I*(*P*) shows an abrupt decrease with pressure at the solution solidification pressure and beyond. The optical density does not exhibit reversibility either, i.e., its initial values are not recovered after pressure release. In addition, the LLSPR band progressively broadens after solidification of the PTM, its initial value increasing up to 40% at 5.5 GPa (about 2.5 GPa of nonhydrostatic pressure). Such a broadening is irreversible, suggesting that partial nanoparticle aggregation takes place. The additional band at 960 nm upon solid-to-liquid transition in downstroke further confirms AuNR aggregation. It has been shown, both experimentally and theoretically, that the appearance of this largely red-shifted band beyond the LLSPR at 680 nm in *AR* = 3 AuNR colloids is an indication of end-to-end aggregated nanorod structures.^19−22^

**Figure 3:**
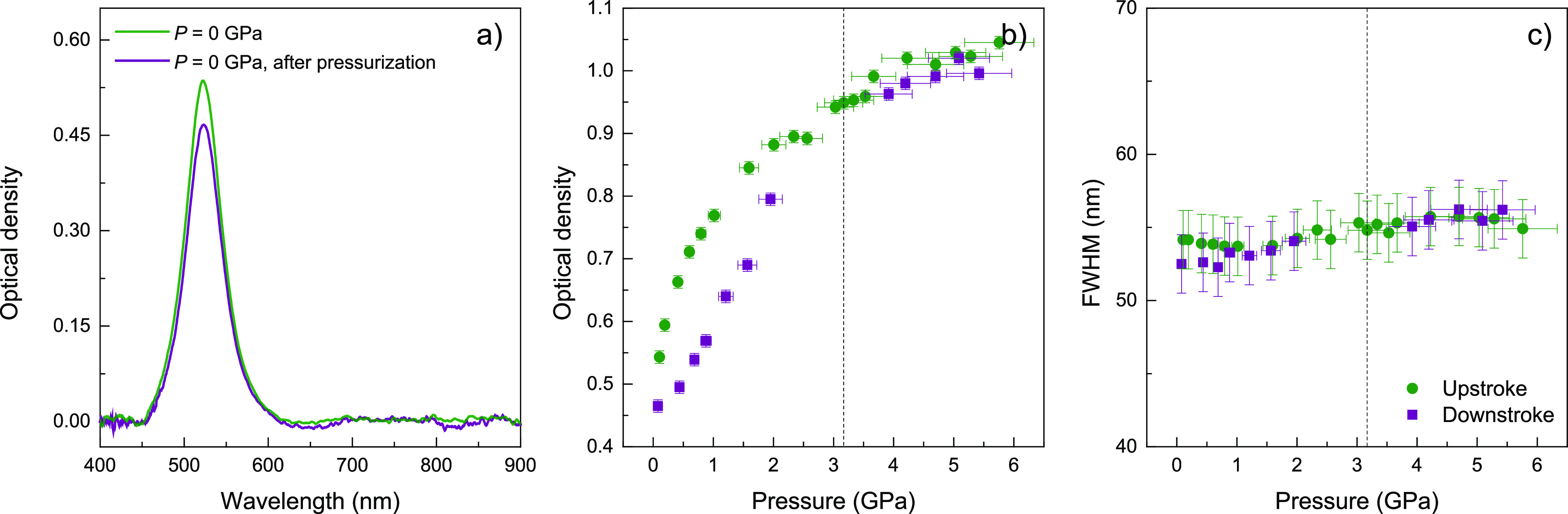
(a) Extinction spectra of 28 nm AuNS in EtOH at ambient pressure, before and after pressurization at 5 GPa. (b) Optical density at the LSPR wavelength as a function of applied pressure. (c) Pressure dependence of the LSPR band fwhm obtained from optical extinction spectra of the AuNS dispersion. Filled circles (green) and squares (purple) correspond to upstroke and downstroke experimental data, respectively. Error bars indicate the standard deviation of each spectral parameter as derived by fitting the LSPR band to a log-normal function. Vertical dashed lines indicate the hydrostaticity limit of the pressure-transmitting medium, as determined from ruby probes.^23,24^

Interestingly, in AuNS colloids in EtOH, neither the LSPR bandwidth nor the optical density undergo significant changes, even after losing the hydrostaticity of the solution, upon solvent solidification around 3 GPa (Figure 3). Once the colloid transits to the liquid state in the downstroke, the optical density at the LSPR frequency progressively decreases toward smaller values (about 10%) with respect to the upstroke values. This behavior likely indicates that AuNS hardly undergo any partial aggregation under the solid-to-liquid transition in downstroke. The degree of aggregation is not significant according to the pressure dependence of the LSPR bandwidth, revealing that AuNS are less prone to aggregate or undergo irreversible deformation induced by the uniaxial stresses associated with solvent solidification. In summary, AuNS maintain their shape and monodispersity during pressure treatments. This result is noteworthy because, at variance with nanorods, it shows that AuNS are well suited for sensing on the basis of single nanoparticle response. To validate this structural scenario, we performed SAXS measurements under identical high-pressure conditions to those in the optical experiments.

### 2.2 Small-Angle X-ray Scattering by Gold Colloids at High Pressure

**Figure 4:**
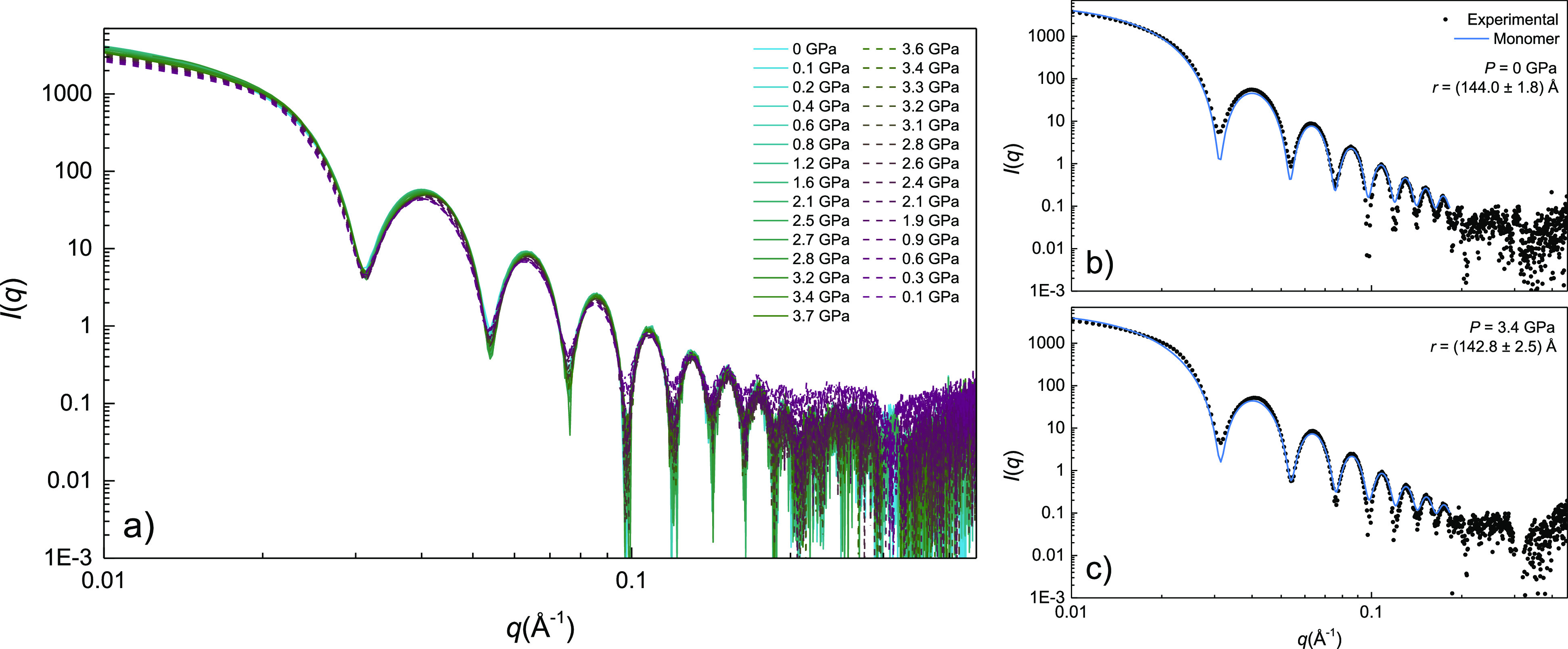
(a) SAXS *I*(*q*) patterns from a dispersion of 28 nm AuNS in ethanol as a function of pressure in the 0–4 GPa range. Solid and dashed lines correspond to upstroke and downstroke measurements, respectively. (b,c) SAXS *I*(*q*) pattern from 28 nm AuNS for two selected pressures: 0 GPa (hydrostatic) and 3.4 GPa (nonhydrostatic). Filled circles correspond to experimental data, and the solid blue line represents the calculated *I*(*q*) curve for a monodisperse (noninteracting) NP structure factor (*S*(*q*) = 1). Magnified (b,c) plots are shown in Figures S1 and S2 in Supporting Information.

Figure 4 shows the variation with pressure of the isotropic SAXS *I*(*q*) pattern of a 28 nm AuNS colloid in EtOH, in both upstroke and downstroke. At ambient pressure, *I*(*q*) can be accurately described in terms of a distribution of AuNS with a form factor corresponding to a hard homogeneous sphere (eq 1). We find no signal associated with interparticle correlation effects, *S*(*q*) = 1, within the available *q* range attained in the DAC (0.01–0.4 Å^–1^). The sphere radius and its standard deviation were derived by fitting *I*(*q*) to the sphere form factor and size distribution (polydispersity) by means of the software SASfit,^25^ through the expressions:
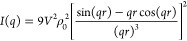
1
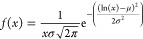
2where *I*(*q*) is the form factor of a sphere of radius *r*, *V* and ρ_0_^2^ are the sphere volume and the electronic density difference between gold and solvent, respectively, *f*(*x*) is the log-normal distribution function of spheres of radius *x*, with *r* = *e*^μ^ the mean sphere radius (the median of the distribution), and σ is the standard deviation (polidispersity).

The fitted sphere radius at ambient conditions is *r* = 14.4 nm (σ = 1.8 nm), a value fully consistent with the mean AuNS diameter determined by TEM, *d* = 28.2(0.4) nm (see Figures 1 and 4b). The *I*(*q*) analysis confirms the monodispersity of the colloid of nonaggregated single spheres, being stable over the whole measured pressure range in both upstroke (hydrostatic and nonhydrostatic ranges) and downstroke. No significant trace of nanoparticle aggregation was observed in any of the *I*(*q*) patterns, even under nonhydrostatic conditions, as it is evident from Figure 4c, in aggreement with the optical extinction spectroscopy results of Figure 3.

**Figure 5:**
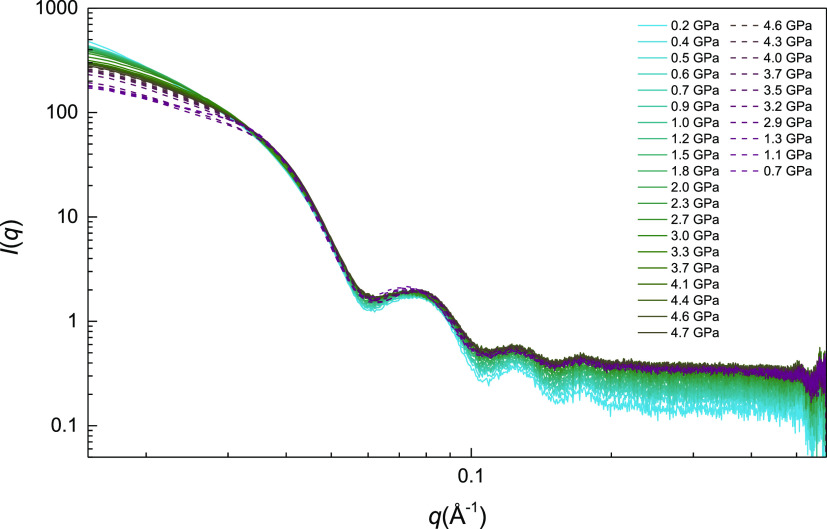
Isotropic SAXS *I*(*q*) patterns of a dispersion of 13 nm diameter, *AR* = 3.0 AuNR in ethanol, as a function of pressure in the 0–5 GPa range. Solid and dashed lines correspond to upstroke and downstroke measurements, respectively.

**Figure 6:**
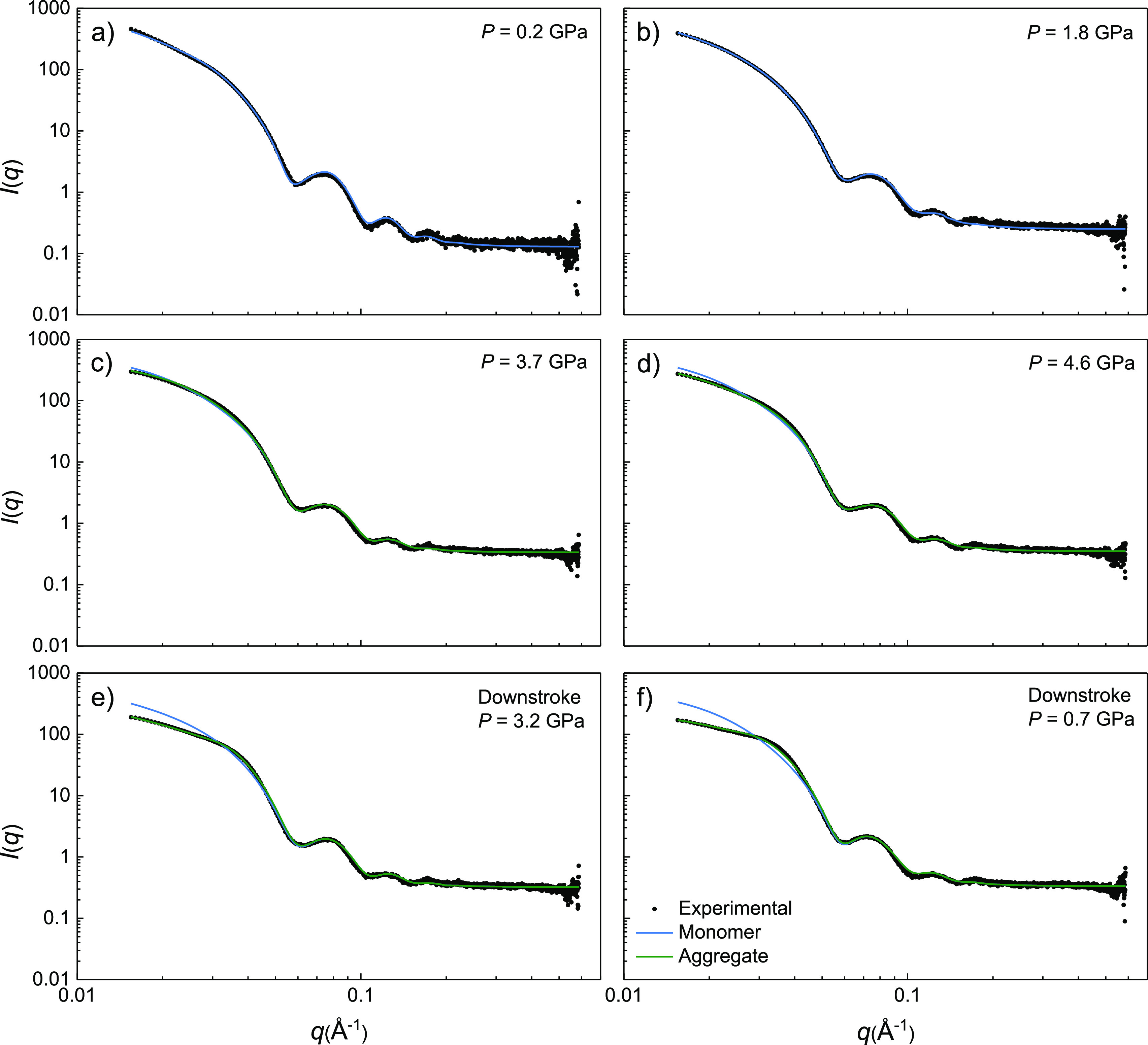
SAXS *I*(*q*) patterns from *AR* = 3.0 AuNR for selected pressures in the hydrostatic range (a,b), nonhydrostatic range (c,d), and in downstroke (e,f). Filled circles correspond to experimental data, and blue and green lines represent the calculated *I*(*q*) curves for a monomer (*S*(*q*) = 1) and partially aggregated-nanoparticle structure factor (*S*(*q*) ≠ 1), respectively. Magnified plots (a–f) are shown in Figures S3–S8 in the Supporting Information.

Figure 5 shows the evolution with pressure of the radial averaged isotropic SAXS *I*(*q*) intensity of the 13 nm-diameter *AR* = 3.0 AuNR colloid in EtOH. In the hydrostatic region, *I*(*q*) can be properly fitted to a fully dispersed colloid (nonaggregated single particles: *S*(*q*) = 1). By assimilating the nanorods to cylinders (Figure 6a,b), we obtain a rod length and radius of *l* = 37.4 nm (σ = 1.2 nm) and *r* = 6.5 nm (σ = 0.6 nm) at 0.2 GPa, respectively. Within the method accuracy, these values are fully consistent with the mean nanoparticle length and diameter, 39(2) × 13.1(5) nm^2^, extracted by TEM (see Figure 1). The nonaggregated behavior observed in the initial AuNR dispersion remains with increasing pressure within the hydrostatic range of the PTM, up to 2.7 GPa. However, under nonhydrostatic conditions (*P* > 2.7 GPa), we observe a remarkable decrease in intensity at low *q* as pressure increases. Interestingly, this decrease becomes even more pronounced once the pressure is released in downstroke measurements. The shape of the measured SAXS patterns at low *q* clearly suggests interparticle correlation effects *S*(*q*) ≠ 1. Such deviations can be interpreted in terms of large-scale aggregates, thus indicating that partial aggregation of the nanoparticles has occurred under nonhydrostatic, high-pressure conditions.

The analysis of *I*(*q*) in both hydrostatic (liquid PTM) and nonhydrostatic (solid PTM) regimes was performed by considering that the AuNR can be present either as a noninteracting nanoparticles (*S*(*q*) = 1) or aggregated to each other (*S*(*q*) ≠ 1). In the hydrostatic range, *I*(*q*) is well accounted for using the expression:
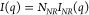
3where *N*_*NR*_ is the number of isolated nanorods, structure factor *S*(*q*) = 1, and *I*_*NR*_(*q*) is the square modulus of the nanoparticle form factor at pressure *P* (see Figure 6a,b). In the nonhydrostatic range in upstroke and at all pressures in downstroke, *I*(*q*) was found to deviate significantly from the isolated nanorod signal *I*_*NR*_(*q*). In such cases, we simulated *I*(*q*) as follows:
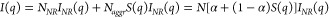
4where α is the fraction of isolated nanorods and (1 – α) is the fraction of aggregated nanorods, on the assumption that isolated and aggregated nanorods have the same form factor. The structure factor *S*(*q*) was determined by fitting the experimental *I*(*q*) data to a Percus–Yevick-type *S*(*q*) function with a packing ratio η = 0.2^26^ (see Figure S9 in Supporting Information). The structure factor *S*(*q*) has a maximum located at *q*_*G*_ = 0.038 Å^–1^, correlation distance of 16.5 nm, and it provides the overall best *S*(*q*) function empirically, accounting for all experimental *I*(*q*) patterns.

The simulations of the aggregation model are summarized in Figure 6c–f, which shows representative SAXS patterns, both in upstroke and downstroke, together with the corresponding simulations, considering both a fully dispersed colloidal solution and the presence of a fraction of aggregated nanoparticles. It should be noted that the same *S*(*q*) function was used in the simulations for all pressures; the only pressure-dependent parameters are *I*_*NR*_(*q*) and α. Although *S*(*q*) may vary with the size of the aggregate (very low *q* range), we used the same *S*(*q*) profile throughout the whole analysis because it provides a suitable description of all *I*(*q*) patterns in the explored pressure range.

**Figure 7:**
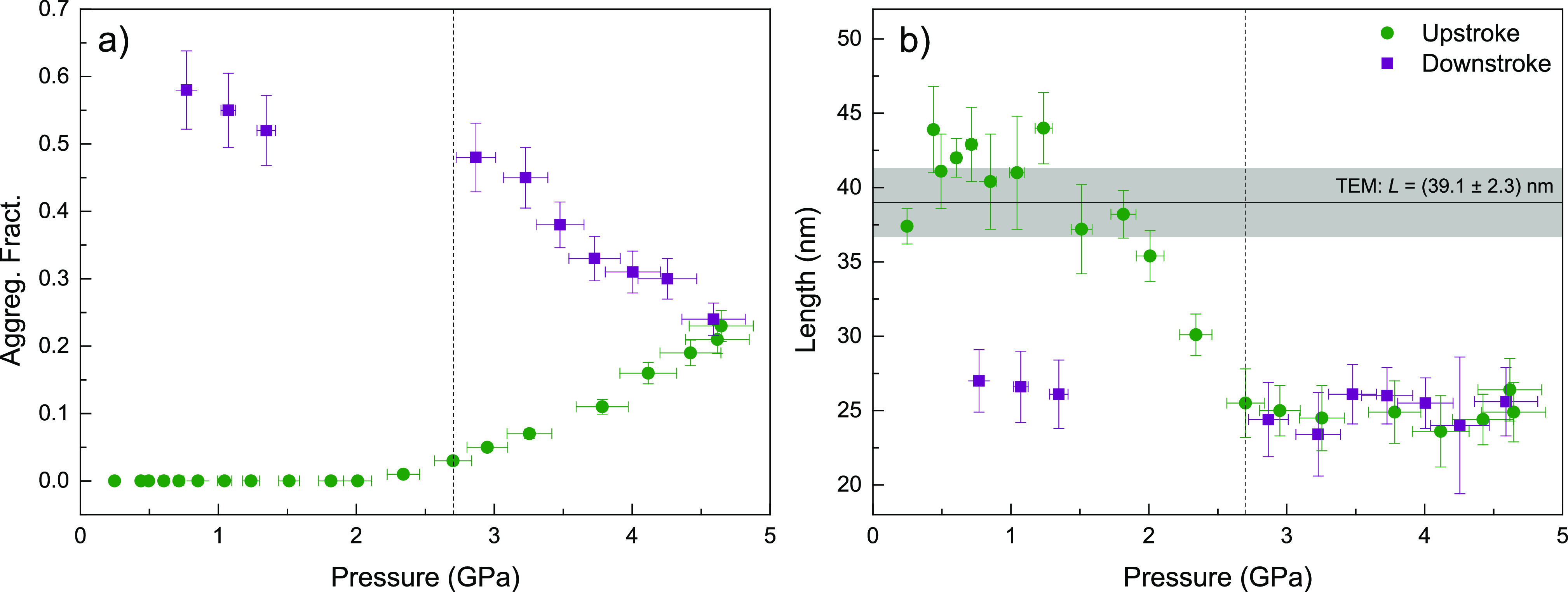
Pressure dependence of the SAXS-derived aggregated nanoparticle fraction (a) and rod length (b). Filled circles (green) and squares (purple) correspond to upstroke and downstroke experimental data, respectively. Gray area indicates the rod length and its standard deviation at ambient pressure according to TEM observations. Vertical dashed lines indicate the transition from liquid-to-solid and solid-to-liquid in upstroke and downstroke, respectively.

From this model, we can estimate the pressure-induced fraction of aggregated nanoparticles as a function of pressure (Figure 7a). The results reveal that nanorods remain stable in the colloid while in the hydrostatic range, i.e., nonaggregated single nanoparticles, as previously evidenced through optical extinction spectroscopy (Figure 2). However, around the solvent solidification pressure, the fraction of aggregated nanoparticles increases progressively with pressure, unveiling the nonhydrostaticity of the solvent as the trigger for colloidal instability under pressure. Specifically, at 4.7 GPa, the maximum pressure reached in these experiments, 2 GPa beyond the solidification pressure at 2.7 GPa, the percentage of aggregated nanorods reaches 20%. Interestingly, once the pressure is released from 4.7 GPa, the fraction of aggregated nanoparticles increases further, up to 60%. According to the obtained structure factor *S*(*q*), the correlation distance better describing the experimental *I*(*q*) data is *R*_*G*_ = 16.5 nm, which fairly corresponds to a nearly side-to-side AuNR configuration as the dominant aggregation mode under high pressure (Figure S9 in Supporting Information). Given that the obtained *S*(*q*) structure factor corresponds to a packing ratio η = 0.2,^27^ the nanorods forming aggregates would be packed with an efficiency of 20%. Besides the aggregate size and other shape effects, this packing ratio is consistent with one-dimensional side-to-side clusters, i.e., one nanorod is displaced vertically with respect to the two neighboring nanorods, and some of them end-to-end linked to other AuNRs. These two aggregation structures coherently account for both SAXS and optical measurements and agree with correlation studies between the type of aggregates and plasmonic effects, reported elsewhere.^19−22^

Notably, the quality of the measured *I*(*q*) patterns allows us to follow the evolution of the AuNR dimensions under the application of pressure, through the analysis of the form factor. Figure 7b shows the pressure dependence of the fitted AuNR length. The results reveal a reduction of 35% in the mean rod length, which takes place around the pressure solidification of the colloid. Our data suggest that the uniaxial stresses derived from solidification of the colloid have a shear effect on the nanorods, breaking them into smaller particles. The present in situ exploration through SAXS confirms previous results on this effect unravelled through TEM observations on recovered pressurized samples, as reported elsewhere.^15^ On the assumption that nanorods break mostly in half, we estimate that the number of rods which underwent plastic deformation was about 60% of the total number of rods.

An intriguing result concerns the observation of pressure-induced aggregation being triggered along with solidification of the solvent. Aggregation increases further with higher applied pressure, and even continues after pressure release in downstroke. This phenomenon resembles the clustering and subsequent precipitation of PEG-capped AuNR when cooling a colloidal solution at 10–20 °C below room temperature. The phenomenon has been associated with conformational changes undergone by the PEG chains in the colloid,^28^ hindering their electrosteric properties and eventually leading to AuNR clustering. Although a similar phenomenon can be invoked for explaining AuNR aggregation under nonhydrostatic pressure conditions, a remarkable difference between temperature- and pressure-induced AuNR aggregate formation is the state of the colloidal solution. While AuNR can diffuse below room temperature in the liquid state, here we demonstrate that AuNR diffusion and aggregation also occurs in the solidified colloidal dispersion.

A plausible explanation for such an unexpected AuNR diffusion through the solidified solvent may be the decrease, by about an order of magnitude, of the dielectric constant of ethanol along with the liquid to solid transition.^29^ The notable decrease of the low-frequency dielectric constant upon solidification implies that the electric-field induced orientation of ethanol molecules is much less pronounced in the solid as compared to the liquid. This effect, together with the loss of electrosteric properties of PEG ligands due to conformational changes upon solidification, can result in aggregation. It should be noted that AuNR diffusion is still possible when ethanol is solidified, considering previous observations that AuNR can diffuse in liquid crystals^30^ and even in mesoporous silica.^31^ Grain boundaries are expected to be preferred sites for aggregation because these regions are the last ones to transit from liquid ethanol to solid ethanol, during the solidification process. The observed AuNR aggregation constitutes clear evidence of AuNR diffusion in solidified ethanol and suggests diffusion processes of PEG-capped AuNR in solid solvents, a process most often ruled out in the realm of nanoscience and nanotechnology.

## 3 Conclusions

We have demonstrated that the stability of gold nanoparticle colloids (28 nm nanospheres and 39 nm × 13 nm^2^ nanorods) under high pressure varies depending on the hydrostaticity of the PTM. We showed that AuNS are suitable for plasmonic sensing at high pressure because they remain isolated (not aggregated) in both the hydrostatic and nonhydrostatic regimes of the PTM, at variance with AuNR. Therefore, single nanoparticle plasmonics can be efficiently used for pressure sensing with AuNS, even under nonhydrostatic conditions. By contrast, AuNR showed a significant tendency to aggregate above the solidification pressure, increasing the fraction of aggregated nanoparticles with pressure. The nanorod size distribution indicates that a NP fraction of 60% undergoes plastic deformation, mostly breaking into two smaller rods due to the combined effect of aggregation and axial stresses in the nonhydrostatic regime. The aggregation process is continuous irreversibly upon pressure release, indicating the inability of the surfactant (capping PEG) to keep the nanoparticles separated in the colloid. This effect has important consequences in the plasmonics of nanorods above the solidification pressure, rendering the AuNR colloid inadequate for sensing under nonhydrostatic conditions.

## 4 Experimental Section

### 4.1 Nanoparticle Synthesis

Chemicals: Gold(III) chloride trihydrate (HAuCl_4_·3H_2_O, ≥99%), hexadecyltrimethylammonium bromide (CTAB, ≥99%), sodium borohydride (NaBH_4_), hexadecyltrimethylammonium chloride (CTAC, 25 wt % in water), benzyldimethylhexadecylammonium chloride (BDAC), ascorbic acid (AA, ≥99%), hydroquinone (HQ, ≥99%), silver nitrate (AgNO_3_, ≥98%), *O*-[2-(3-mercaptopropionylamino)ethyl]-*O*′-methylpolyethylene glycol (PEG-SH, Mw: 5K) were purchased from Sigma-Aldrich. Ethanol and methanol were purchased from Scharlab. All chemicals were used without further purification. Milli-Q water (resistivity 18.2 MΩ·cm at 25 °C) was used in all experiments. All glassware was cleaned with aqua regia, rinsed with Milli-Q water, and dried before use.

Synthesis of single-crystalline AuNS and AuNR: Single-crystalline AuNS and AuNR were synthesized via well-established seeded-growth methods.^32,33^ First, gold seeds (∼1.5 nm) were prepared by fast reduction of HAuCl_4_ (5 mL, 0.25 mM) with freshly prepared NaBH_4_ (0.3 mL, 10 mM) in aqueous CTAB solution (100 mM) under vigorous stirring for 2 min at room temperature and then kept undisturbed at 27 °C for 30 min to ensure complete decomposition of sodium borohydride. The mixture turns from light yellow to brownish indicating the formation of gold seeds. To grow 12 nm gold nanospheres from gold seeds, an aliquot of seed solution (0.6 mL) was added under vigorous stirring to a growth solution containing CTAC (100 mL, 100 mM), HAuCl_4_ (0.36 mL, 50 mM), and ascorbic acid (0.36 mL, 100 mM). The mixture was left undisturbed for 12 h at 25 °C. The solution containing gold nanoparticles was centrifuged (9000 rpm for 1 h) to remove excess of CTAC and ascorbic acid and redispersed in CTAB 1 mM to a final gold concentration of 1 mM.

To grow 12 nm AuNS up to 28 nm diameter, an aliquot of 12 nm AuNS solution (2.14 mL, 1 mM) was added under magnetic stirring to a growth solution (100 mL) containing BDAC (100 mM), HAuCl_4_ (0.25 mM), and ascorbic acid (0.5 mM). The mixture was left undisturbed for 30 min at 30 °C and then washed twice by centrifugation (8000 rpm for 1 h). The particles were finally dispersed in 1 mM CTAB to yield a final gold concentration equal to 1 mM.

AuNR were synthesized as described elsewhere^33^ with some modifications. AuNR were prepared by adding an aliquot of gold seeds (∼1.5 nm, 1 mL) under vigorous stirring to a growth solution containing CTAB (100 mL, 100 mM), HAuCl_4_ (1 mL, 50 mM), HQ (15 mL, 100 mM), and AgNO_3_ (1.4 mL, 10 mM). The stirring was stopped after 5 min, and the mixture was left undisturbed for 2 h at 30 °C. The nanoparticles were washed by two centrifugation rounds (8000 rpm, 30 min) to remove excess reagents. After the second centrifugation step, the solution was redispersed in CTAB (100 mM) to a final gold concentration of 1 mM. AuNR (15 mL, 1 mM) were partially oxidized with Au^3+^ (3 mL, 1 mM, 1 mL/h) until the longitudinal absorption band was located at 687 nm. Then, the solution was centrifuged (9000 rpm for 1 h) and redispersed in CTAB 1 mM. The concentration of gold for ligand exchange was 1 mM.

Ligand exchange:^34^ To replace the surfactant and transfer the gold nanoparticles to alcoholic mixture, thiolated polyethylene glycol (PEG-SH) of molecular weight of 5K was used. An aqueous solution of PEG-SH (10.9 mg for 28 nm AuNS, and 21.3 mg for AuNR, dissolved in 2 mL of water) dispersion was added dropwise under stirring to a dispersion of gold nanoparticles (12 mL, 1 mM) in CTAB 1 mM. The solution was left for 2 h under stirring and then centrifuged twice in ethanol. Pegylated gold nanoparticles were finally dispersed in ethanol.

Representative TEM images and extinction spectra of the AuNP colloids employed in the experiments are shown in Figure 1. The investigated AuNS have an average diameter of 28.2 ± 0.4 nm, and their extinction spectrum shows the characteristic LSPR band centered at 523 nm. AuNR have a mean length of 39.1 ± 2.3 nm, mean diameter of 13.1 ± 0.5 and an *AR* distribution 3.0 ± 0.2, and the optical spectrum shows the characteristic band structure associated with a transversal LSPR at 510 nm and a longitudinal LSPR at 687 nm.

### 4.2 Optical Extinction Spectroscopy at High Pressure

High-pressure experiments were carried out in a Boëhler-Almax DAC equipped with ultralow-fluorescence diamond IIa anvils with 350 μm diameter culets. The 200 μm thick Inconel gaskets were preindented to 40–50 μm and drilled with 150 μm diameter holes with a BETSA motorized electrical discharge machine to create the hydrostatic chamber. The DAC was loaded with EtOH AuNP solutions and several ruby microspheres (10–30 μm diameter) as pressure probes.^23,24^ The solution itself acted as the pressure-transmitting medium. The hydrostatic pressure range and liquid–solid pressure transition of AuNP solutions were determined from the pressure dependence of the full width at half-maximum (fwhm) of the ruby emission R lines, whereas the pressure inside the cavity was determined from the R_2_ line shift following the accepted pressure dependence established elsewhere.^35^

Optical extinction spectra under high-pressure conditions were collected on a home-built fiber-optic-based microscope,^36^ equipped with two Cassegrain 20× reflecting objectives mounted on two independent *x*-*y*-*z* translational stages for the microfocus beam, the objective lens, and a third independent *x*-*y* translation stage for the DAC holder. Spectra in the ultraviolet–visible and near-infrared ranges were recorded with two spectrometers, an Ocean Optics USB 2000 and a NIRQUEST 512, employing Si- and InGaAs-CCD detectors, respectively. The ambient pressure spectra taken upon pressure release were collected in the diamond anvil cell. The pressure was slowly released until a small air bubble was formed inside the cavity. Then, the cavity was sealed again and checked that the ruby remained at ambient pressure. The extinction spectrum was collected right after sealing and ca. 10 min later; the obtained spectra were reproducible within the spectral accuracy. The *I* and *I*_0_ transmitted intensities were measured in two separate experiments with the same DAC by loading it first with the AuNP solutions (*I*) and then with the corresponding solvent (*I*_0_), covering the same pressure range.

### 4.3 Small-Angle X-ray Scattering at High Pressure

SAXS measurements were performed on the SWING beamline, at the SOLEIL Synchrotron. The beamline was adapted to high-pressure SAXS by inserting a membrane diamond anvil cell equipped with 1 mm-thick, 3 mm-diameter anvils ground with 0.8 mm culets (see Figure S10 in the Supporting Information). This anvil geometry allowed us to work with gasket cavities of 300 μm diameter, which properly fit onto the 200 × 150 μm^2^ X-ray beam spot and attenuate the beam intensity to only 20% at the working energy. These conditions are crucial for obtaining suitable SAXS signals, *I*(*q*), for structural analysis within the 0–5 GPa pressure range. The experiments were carried out employing a monochromatic X-ray beam of 0.8265 Å passing through the DAC and focused at the two-dimensional EIGER-4 M detector position, located 2061.02 mm downstream the sample. The selected sample–detector distance and beam energy (15 keV) allowed us to locate the optimum scattering angular range, in order to obtain the most precise values of the form factor (size and shape of the NP) and the structure factor (aggregate formation or NP precipitation). The gold colloids were loaded onto a membrane DAC with automatic control over the membrane pressure, 800 μm culet diameter diamonds, and a 300 μm drilled hole in a CuBe gasket, preindented to 100 μm. Ruby microspheres of 10–20 μm diameter were placed into the sample chamber as pressure markers, following the relationship between R_1,2_-line shift and pressure.^35^ The hydrostaticity of the pressure-transmitting medium was monitored through the ruby R-line broadening, whose line width is known to slightly decrease with pressure in the hydrostatic range and progressively broaden with pressure in the nonhydrostatic range.^23,24^ The relatively large size of the diamonds enabled us to load a significant amount of sample (0.1 mm^3^). However, it also limits the achievable pressure range to 5 GPa. In these experiments, we worked with AuNP colloids in EtOH as PTM, since it solidifies at about 3.5 GPa, thus enabling us to explore the effects of both hydrostatic and nonhydrostatic pressure on colloidal stability. SAXS images with 1 s exposure time were normalized and azimuthally integrated into curves using the local application Foxtrot, then further analyzed with the SASfit and SasView softwares^25,37^ to test the geometries corresponding to each colloid and to explore different structure factors related to NP aggregation.

## Supporting Information Available

(1) Experimental and calculated SAXS intensity *I*(*q*) for AuNS colloidal dispersions; (2) experimental and calculated SAXS intensity *I*(*q*) for AuNR colloidal dispersions; (3) structure factor *S*(*q*); (4) SAXS experimental setup (PDF)

## Abbreviations

AuNR: gold nanorods
AuNS: gold nanospheres
AuNP: gold nanoparticle
PEG: thiolated polyethylene glycol
EtOH: ethanol
DAC: diamond anvil cell
LLSPR: longitudinal localized surface plasmon resonance
LSPR: localized surface plasmon resonance
PTM: pressure transmitting medium
SAXS: small-angle X-ray scattering
TEM: transmission electron microscopy
