# Supplementary material for: Behavior of Au Nanoparticles under Pressure Observed by In Situ Small-Angle X-ray Scattering
Source: ACS Nano. 2022 Dec 16;17(1):743–51. doi: 10.1021/acsnano.2c10643 (PMC9835983; doi:10.1021/acsnano.2c10643)
Supplement: Supplementary file 1 — nn2c10643_si_001.pdf [file nn2c10643_si_001.pdf]

Supporting Information

Behaviour of Au Nanoparticles under Pressure Observed by *In-Situ* Small-Angle X-Ray Scattering

Camino Martín-Sánchez[a,b,*], Ana Sánchez-Iglesias[c], José Antonio Barreda-Argüeso[a], Alain Polian[d,e], Luis M. Liz-Marzán[c,f,g], Fernando Rodríguez[a]

[a] MALTA Consolider, Departamento CITIMAC, Facultad de Ciencias, Universidad de Cantabria, Santander 39005, Spain

[b] Faculté des Sciences, Département de Chimie Physique, Université de Genève, 30 Quai Ernest-Ansermet, CH-1211 Genève, Switzerland

[c] CIC biomaGUNE, Basque Research and Technology Alliance (BRTA), Paseo de Miramón 194, Donostia-San Sebastián, 20014, Spain

[d] Synchrotron SOLEIL, L'Orme des Merisiers St.Aubin, BP48, 91192 Gif-sur-Yvette, France

[e] Sorbonne Université, UMR CNRS 7590, Institut de Minéralogie, de Physique des Matériaux et de Cosmochimie, IMPMC, 75005 Paris, France

[f] Ikerbasque, Basque Foundation for Science, Bilbao, 43018, Spain

[g] Centro de Investigación Biomédica en Red, Bioingeniería, Biomateriales y Nanomedicina (CIBER-BBN), Paseo de Miramón 194, Donostia-San Sebastián, 20014, Spain

Supporting Information content:

1) Experimental and calculated SAXS intensity $I(q)$ for gold nanospheres (AuNS) colloidal dispersions.

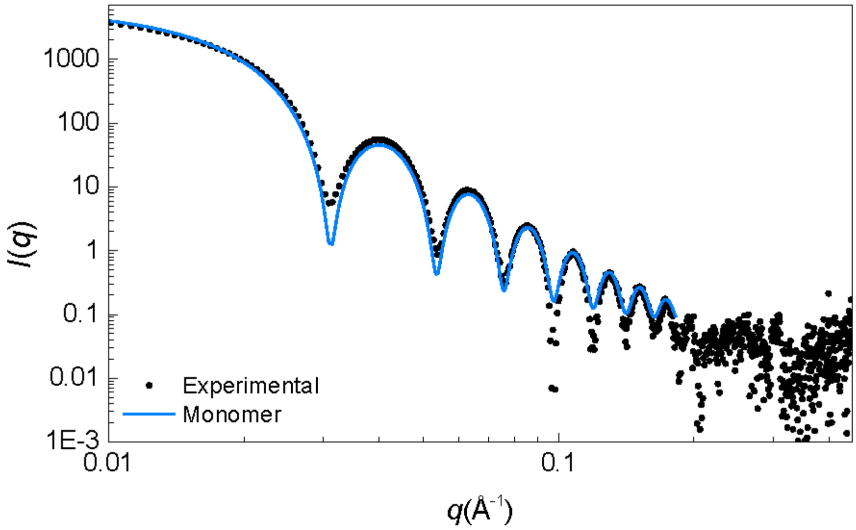

Figure S1. Experimental (filled circles) and calculated (solid blue line) SAXS intensity $I(q)$ for AuNS colloid in ethanol at 0 GPa in the hydrostatic range.

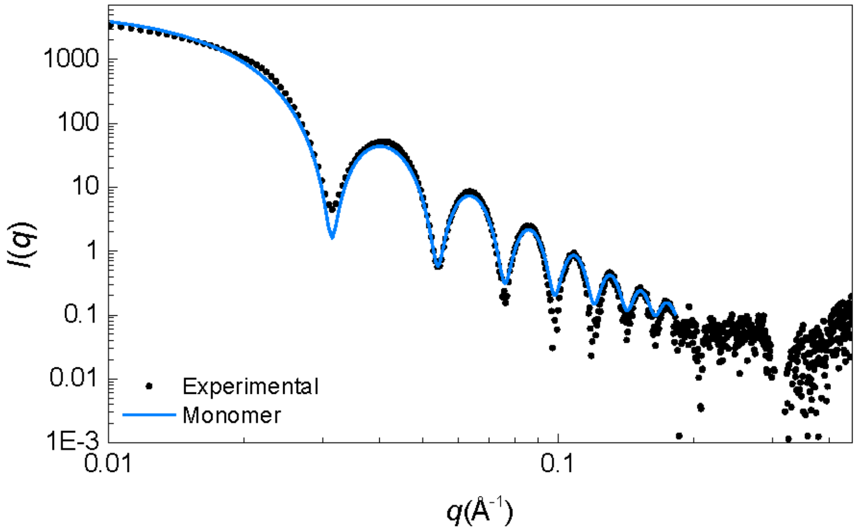

Figure S2. Experimental (filled circles) and calculated (solid blue line) SAXS intensity $I(q)$ for AuNS colloid in ethanol at 3.4 GPa in the non-hydrostatic range (solidification pressure: P_{sol} = 2.7 GPa).

2) Experimental and calculated SAXS intensity $I(q)$ for gold nanorods (AuNR) colloidal dispersions.

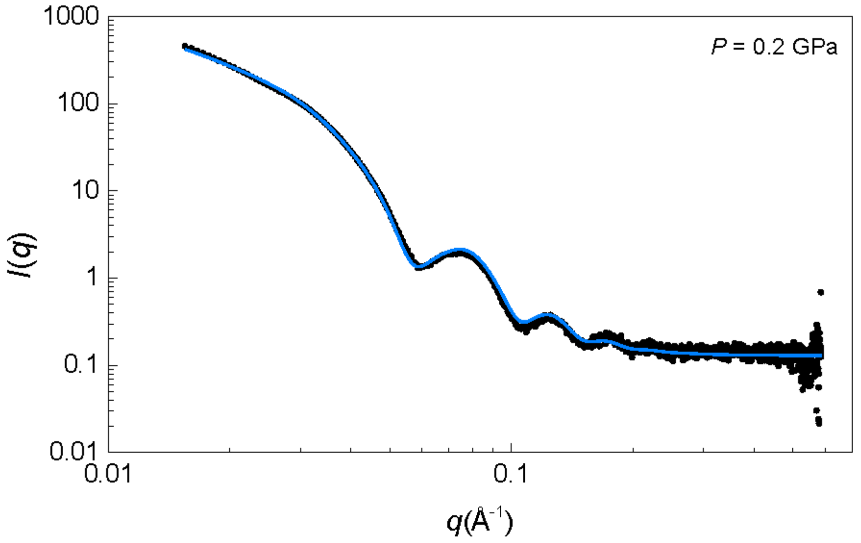

Figure S3. Experimental and calculated SAXS intensity $I(q)$ for AuNR colloids in ethanol at 0.2 GPa in the hydrostatic range in upstroke (solidification pressure: P_{sol} = 2.7 GPa).

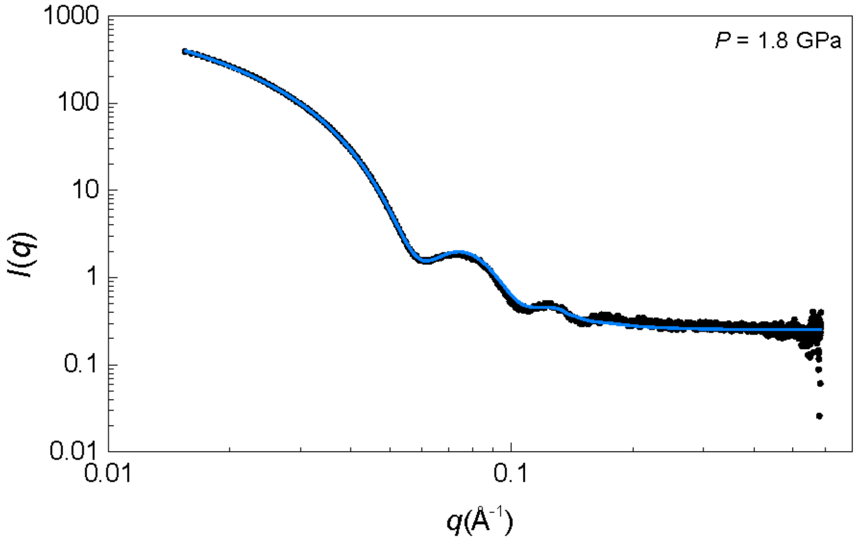

Figure S4. Experimental and calculated SAXS intensity $I(q)$ for AuNR colloids in ethanol at 1.8 GPa in the hydrostatic range in upstroke (solidification pressure: P_{sol} = 2.7 GPa).

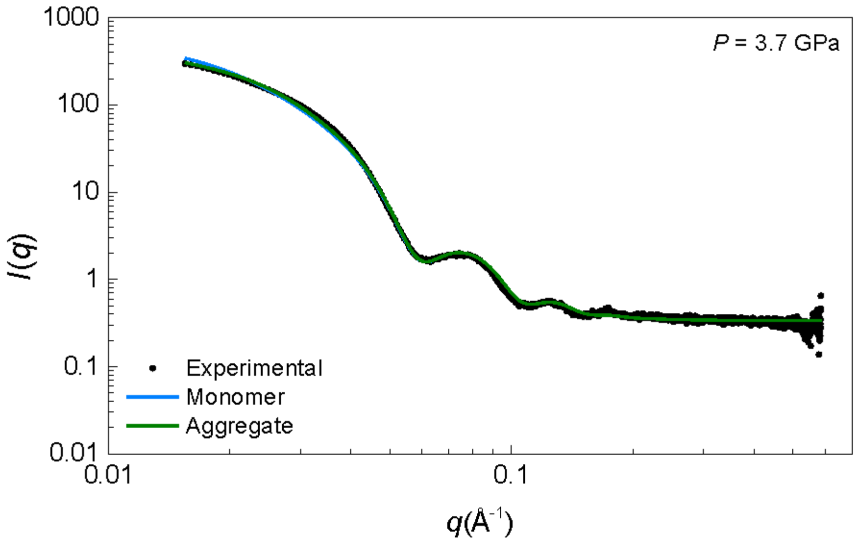

Figure S5. Experimental and calculated SAXS intensity $I(q)$ for AuNR colloids in ethanol at 3.7 GPa in the non-hydrostatic range in upstroke (solidification pressure: P_{sol} = 2.7 GPa).

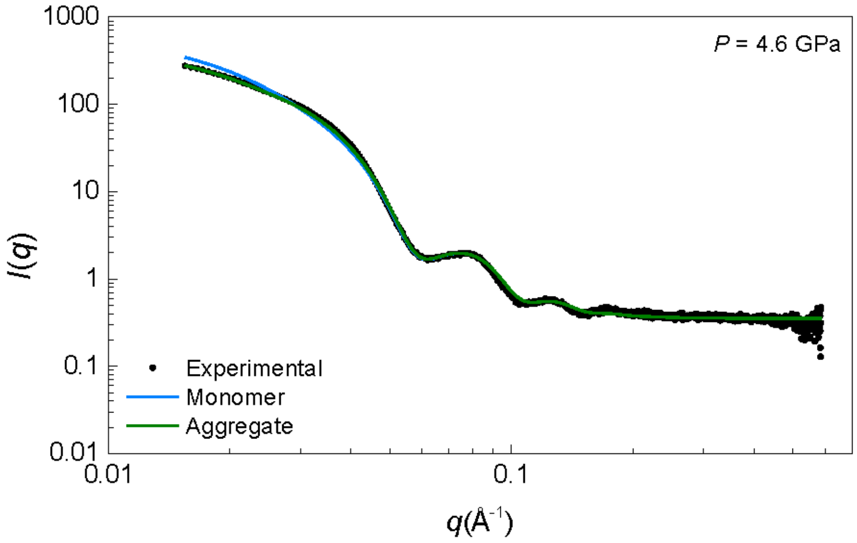

Figure S6. Experimental and calculated SAXS intensity $I(q)$ for AuNR colloids in ethanol at 4.6 GPa in the non-hydrostatic range in upstroke (solidification pressure: P_{sol} = 2.7 GPa).

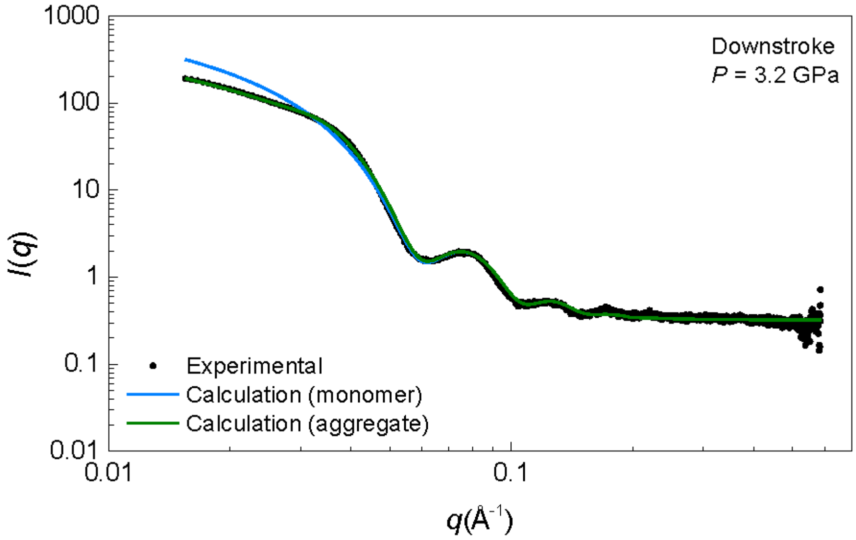

Figure S7. Experimental and calculated SAXS intensity $I(q)$ for AuNR colloids in ethanol at 3.2 GPa in the non-hydrostatic range in downstroke (solidification pressure: P_{sol} = 2.7 GPa).

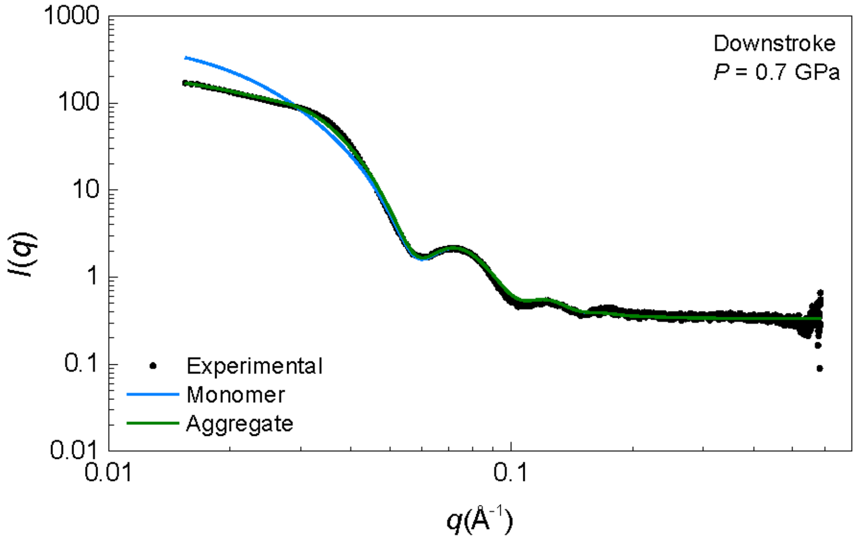

Figure S8. Experimental and calculated SAXS intensity $I(q)$ for AuNR colloids in ethanol at 0.7 GPa in the hydrostatic range in downstroke (solidification pressure: P_{sol} = 2.7 GPa).

3) Structure factor $S(q)$

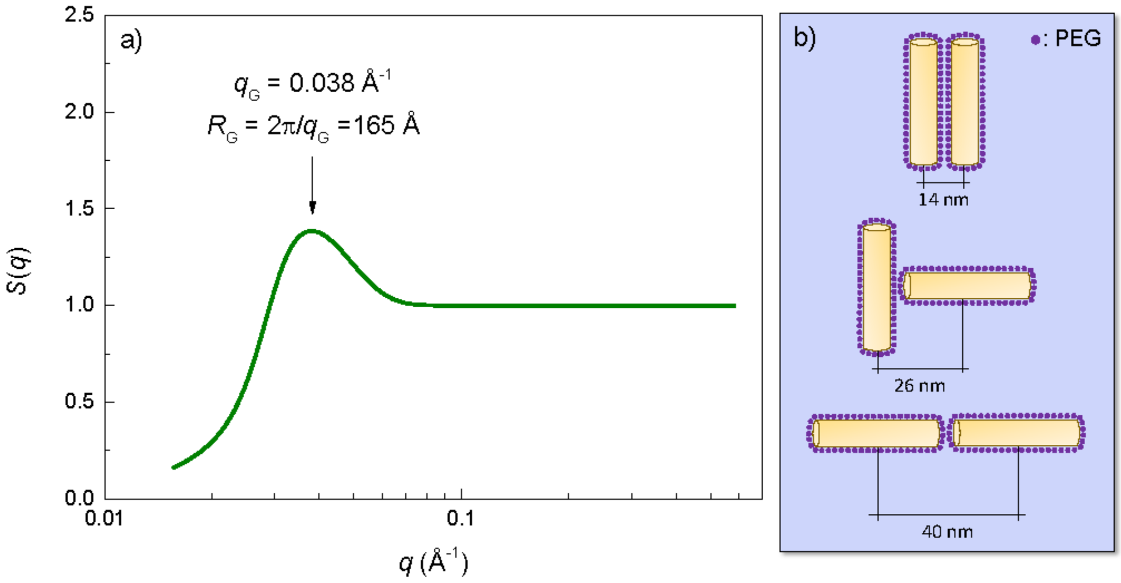

Figure S9. a) Empirical structure factor $S(q)$ derived from $I(q)$. The obtained $S(q)$ curve can be simulated by means of a Percus-Yevick-type correlation function with a packing ratio $\eta = 0.2$ [1,2]. b) Schematic view of possible AuNR aggregation modes: side-to-side, side-to-tail and tail-to-tail. Associated aggregation distance for each mode is indicated.

In order to properly describe the $I(q)$ patterns under non-hydrostatic conditions and in downstroke, it was necessary to model the aggregate presence. To do so, we have considered the following structure factor $S(q)$:

$$S(q) = I_0 q\, e^{-\left(\frac{q_{01}-q}{\beta}\right)^2} + \frac{1}{e^{(q_{02}-q)\gamma}+1} \qquad (1)$$

with $I_0 = 14$; $q_{01} = 0.028$ Å^{-1}; $q_{02} = 0.029$ Å^{-1}; $\beta = 0.02$ Å^{-1}; $\gamma = 350$ Å; where β and γ define the nearest neighbour distance distribution of the aggregate through the $S(q)$ maximum, q_G, as $R_G = 2\pi/q_G$ (Figure S9a). Eq. (1) results in a Percus-Yevick approximation-type [1] structure factor whose maximum is located at $q_G = 0.038$ Å^{-1} - correlation distance of 16.5 nm - (see figure S9b), and it provides the best $S(q)$ function empirically accounting for the experimental $I(q)$ patterns.

4) SAXS experimental setup

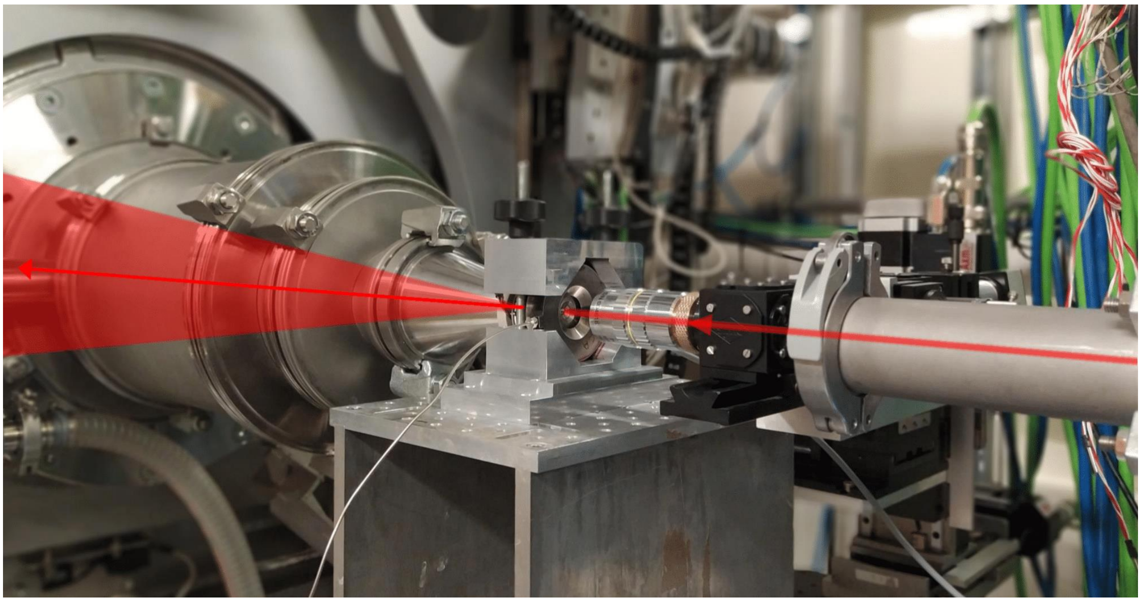

Figure S10. Experimental setup for small-angle x-ray scattering of the Swing beamline at the SOLEIL synchrotron. Red line indicates the incoming x-ray beam passing through the Diamond Anvil Cell. The long-distance objective is placed in front of the DAC –with the x-ray beam shutter off – for measuring the ruby luminescence; *i.e.* pressure marker. It moves out of the x-ray beam during SAXS measurements.

References

[1] Percus, J. K.; Yevick, G. J. Analysis of Classical Statistical Mechanics by Means of Collective Coordinates. *Phys. Rev.* **1958**, 110, 1.

[2] Perram, J. W. Hard Sphere Correlation Functions in the Percus-Yevick Approximation. *Mol. Phys.* **1975**, 30, 1505-1509
